# Going to Bat(s) for Studies of Disease Tolerance

**DOI:** 10.3389/fimmu.2018.02112

**Published:** 2018-09-20

**Authors:** Judith N. Mandl, Caitlin Schneider, David S. Schneider, Michelle L. Baker

**Affiliations:** ^1^Department of Physiology, McGill University, Montreal, QC, Canada; ^2^Department of Microbiology and Immunology, McGill University, Montreal, QC, Canada; ^3^McGill Research Center for Complex Traits, McGill University, Montreal, QC, Canada; ^4^Department of Microbiology and Immunology, Stanford University, Stanford, CA, United States; ^5^Australian Animal Health Laboratory, Health and Biosecurity Business Unit, Commonwealth Scientific and Industrial Research Organisation, Geelong, VIC, Australia

**Keywords:** bats (Chiroptera), viral immunology, host pathogen interaction, disease tolerance, comparative genome analyses, innate immunity

## Abstract

A majority of viruses that have caused recent epidemics with high lethality rates in people, are zoonoses originating from wildlife. Among them are filoviruses (e.g., Marburg, Ebola), coronaviruses (e.g., SARS, MERS), henipaviruses (e.g., Hendra, Nipah) which share the common features that they are all RNA viruses, and that a dysregulated immune response is an important contributor to the tissue damage and hence pathogenicity that results from infection in humans. Intriguingly, these viruses also all originate from bat reservoirs. Bats have been shown to have a greater mean viral richness than predicted by their phylogenetic distance from humans, their geographic range, or their presence in urban areas, suggesting other traits must explain why bats harbor a greater number of zoonotic viruses than other mammals. Bats are highly unusual among mammals in other ways as well. Not only are they the only mammals capable of powered flight, they have extraordinarily long life spans, with little detectable increases in mortality or senescence until high ages. Their physiology likely impacted their history of pathogen exposure and necessitated adaptations that may have also affected immune signaling pathways. Do our life history traits make us susceptible to generating damaging immune responses to RNA viruses or does the physiology of bats make them particularly tolerant or resistant? Understanding what immune mechanisms enable bats to coexist with RNA viruses may provide critical fundamental insights into how to achieve greater resilience in humans.

## Introduction

An estimated ~60% of emerging infectious diseases are caused by pathogens which originate from a non-human animal source, referred to as zoonoses (1–3). Moreover, the frequency of outbreaks caused by zoonotic pathogens has been increasing over time in the human population, with viruses being the most successful at crossing the species barrier (2–4). Given the impact of viral zoonoses on global public health, considerable resources have been invested into better understanding patterns in their emergence to improve predictions of where they might arise. One key variable in such predictions is to determine the animal reservoir populations within which these novel viruses can be maintained indefinitely (with or without disease) and which therefore act as sources for transmission to humans (5). In some instances, epidemiological associations may provide clues to identifying a reservoir host species, and the detection of natural infection through seroconversion or the virus itself provides further evidence. Recently, phylogenetic analyses have also been used to investigate viral origins—with a presence of greater diversity and of strains ancestral to those in humans being indicative of a virus circulating within a particular natural host population (6).

Once identified, viral reservoirs have historically been critical levers through which to reduce human cases (5). However, reservoir hosts may also provide us with fundamental insights into host-pathogen interactions and are a rich opportunity to examine the immunological processes that contribute to patterns governing which pathogens cross into humans, cause disease and why (7, 8). This can be particularly informative as in many instances, the zoonotic viruses that are so pathogenic in humans do not cause disease in the reservoirs with which they coexist.

## Bats are the reservoirs for many human viruses

Bats have been confirmed as reservoir hosts for many viruses, several of which are associated with fatality rates as high as 90% among diagnosed human cases. It has long been appreciated that rabies and other lyssaviruses causing lethal encephalitis can be transmitted from numerous bat species (9, 10). Live Marburg virus (MARV) has been isolated from *Rousettus aegyptiacus* fruit bats which, jointly with epidemiologic evidence and detection of viral RNA, strongly suggests that *R. aegyptiacus* is a reservoir host of this filovirus (11). The related ebolavirus (EBOV) likely also circulates in African fruit bats, with a few species having been implicated so far—the mobility of which accounts for the sudden appearance of Ebola in West Africa during the 2014 outbreak, a region where ebolavirus had not previously been detected (12, 13). The highly pathogenic henipaviruses, of which Hendra virus emerged in Australia and Nipah virus in South-east Asia via horse and pig intermediate hosts respectively, have been shown to be transmitted from *Pteropus* bats (14, 15). In China, horseshoe *Rhinolophus* bats have been identified as the reservoirs for SARS coronavirus via palm civet intermediate hosts, the cause of a large outbreak of atypical pneumonia across several countries that began in 2002 in China (16–18). More recently, MERS coronavirus that has caused lethal respiratory infections mostly in Saudi Arabia, likely transmitted via dromedary camels, was shown to be closely related to several bat coronaviruses, including those sequenced from *Neoromicia capensis, Pipistrellus abramus*, and *Vespertilio superans* bats (19, 20). Moreover, additional viruses may continue to emerge from bats, as in the single case of sosuga virus infection in a wildlife biologist collecting bats in South Sudan (21).

In addition to these emerging zoonotic viruses, bats may be the source of a number of viruses with which humans have older evolutionary associations. For instance, bats harbor viruses closely related to both mumps (rubula virus) and measles (morbilli virus) and have likely been donors of these viruses to other mammalian groups, possibly including humans (6, 22). Furthermore, both Old and New World bats carry diverse hepadnaviruses, some of which are related to hepatitis B virus and can infect human hepatocytes (23). Hepaciviruses that are related to hepatitis C virus and pegiviruses that are related to human GB viruses were detected in the sera of many different bat species, and given the basal position of these bat viruses in phylogenetic trees, may also represent strains ancestral to those found in humans (24, 25).

**Figure 1:**
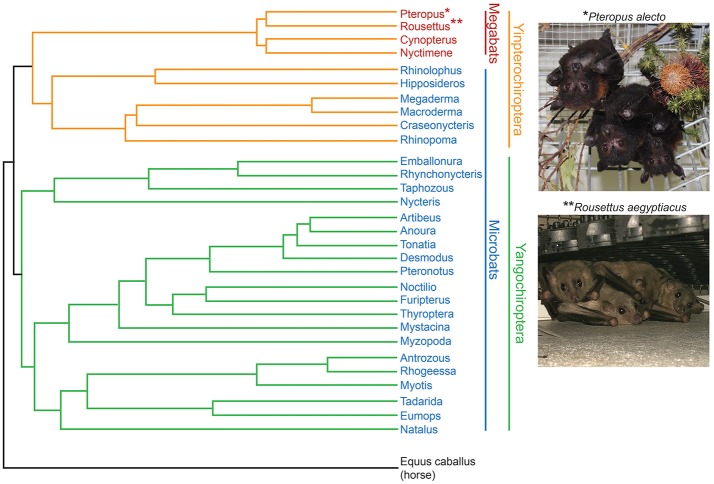
Phylogeny of bat genera [modified from Teeling et al. (31), note that branch lengths are not to scale] indicating the two most studied bat species so far, *Pteropus alecto* and *Rousettus aegyptiacus*. Photos provided by Susanne Wilson, CSIRO, Australia (Pteropus); Anne Balkema-Buschmann and Nils Kiley, Friedrich-Loeffler-Institut, Germany (Rousettus).

The preponderance of links between bat and human pathogens has led to a debate about whether bats disproportionately contribute to emerging viral infections crossing the species barrier into humans (26–30). Given the diversity of the *Chiroptera* order (Figure 1), we may simply see more bat viruses because there are so many (>1,300) species of bats (31). However, even when accounting for the fact that they make up ~20% of extant terrestrial mammals, bats are overrepresented as reservoir hosts of pathogens with a high potential for spilling into human populations (32, 33). In fact, no known predictors that have been described to impact the likelihood of crossing the species barrier, including reservoir host ecology, phylogenetic relatedness to humans or frequency of reservoir-human contact, explain this pattern (32). Thus, why bats are such a frequent source of pathogenic human viruses remains a tantalizing mystery.

Among viruses, those that have genomes encoded by RNA generally jump across species boundaries more frequently, presumably due to their inherently greater mutation rates that facilitate the rapid adaptation to replicating within new hosts (34). Interestingly, all pathogenic viruses that have made the jump to humans for which bat species may be reservoirs share the common feature that they have single-stranded RNA genomes (with the exception of hepadnaviruses which have a DNA genome but replicate via an RNA intermediate). So far, available evidence suggests that bats remain disease-free when infected with the RNA viruses they carry—even those highly pathogenic to humans—and are able to coexist with them without detectable fitness costs using measures such as changes in temperature, loss of body weight, or overt signs of inflammation (35). Indeed, so far only one RNA virus studied which circulates in a bat population has been shown to consistently cause significant morbidity and mortality: tacaribe virus in the Jamaican fruit bat (*Artibeus jamaicensis*), which recent evidence suggests is not a reservoir host for this virus (36). Data from experimental rabies and lyssavirus infections suggests that rhabdoviruses may also cause disease in bats, although experimental infection outcome is very dependent on the infection route. Intracerebral infection with different strains and in different bat species invariably led to death (37, 38). In contrast, intramuscular infection led to muscle weakness, paralysis and visible histological CNS lesions in 30% of experimentally infected flying foxes (*Pteropus poliocephalus*) (39). Similarly, a subset of vampire bats (*Desmodus rotundus*) experimentally infected intramuscularly with a high dose of rabies virus remained healthy despite viral shedding in the saliva and survived (40). Naturally infected bats are thought to either die or remain healthy and seroconvert, but transmission in free-ranging populations remains incompletely understood (41).

While bats seem to be frequent hosts for RNA viruses, current available data indicates that primates and humans disproportionately harbor DNA viruses such as herpesviruses (32). Interestingly, it is these DNA viruses that can persist in an individual which can also be found in isolated, small indigenous groups—perhaps suggestive of humans having a more ancient relationship with such DNA viruses (42). It may even be the case that persistent DNA viruses in humans impact immune responses specifically to RNA viruses, but this has not yet been examined. It is likely that differences in evolutionary history of pathogen exposure between bats and humans have led to distinct adaptations in anti-viral immune responses and the ability to tolerate certain infections without disease while being susceptible to others. Importantly, bats differ in many aspects of their physiology and behavior from humans that may have direct or indirect effects on immune function.

## Bat life history traits

Bats are a monophyletic mammalian group traditionally divided by morphological data into two suborders, the megabats and microbats, which more recent molecular data has revised into the Yinpterochiroptera and Yangochiroptera suborders (Figure 1). Bats possess a suite of traits that make them distinct from other mammals in a number of ways. These unique life history traits may play a role in understanding which pathogens bats have evolved to coexist with and why. In particular, such traits may explain the ability of bat populations to maintain particular viral pathogens indefinitely, and may have effects on immune function through specific energetic or evolutionary trade-offs we have yet to better define.

### Longevity, metabolic rate, and hibernation

Despite the diversity of viruses carried by bats, they are not typically known to cause mass bat die-offs or reduce bats' remarkable longevity. In this respect, bats represent a potential opportunity for long-term persistence of viruses within a population and across generations. Bats live significantly longer than similarly-sized terrestrial mammals and, despite their small size, are characterized as “slow” mammals in the slow-fast continuum (43, 44). Although their weights range from 2 grams to 2 kilograms, with respect to longevity bats group with large mammals such as humans and non-human primates (45). Aerial living has an obvious advantage in avoiding predation, but bats outlive even birds. For example, the Brandt's bat (*Myotis brandtii*) lives up to 41 years, compared to *Selasphorus platycercus*, a bird species of similar size that lives for ~14 years (45, 46). Thus, flight can only partially account for their extraordinarily long lives. Initially, the longevity of some bats was attributed to seasonal hibernation, as temperate-zone species enter continuous torpor of up to 75 days, with a dramatic drop in metabolic rate such that small fat reserves can sustain them throughout the entire hibernating season (43). However, even non-hibernating bat species live three times longer, on average, than predicted by their size, and heterothermy is not an accurate predictor of lifespan in other mammalian orders, suggesting that the driving force behind their surprising longevity is intrinsic to bats as a group (47–49).

Like other “slow” mammals, bat females typically only have one offspring per year, perhaps because the volant lifestyles of bats make it difficult to rear more than one offspring, as pregnant females and those with recent births must navigate and forage with added weight; on average, neonatal bat pups are ¼ of their mother's weight (50). The physical and energetic constraints of rearing multiple offspring may necessitate small litters, which would in turn require prolonged reproductive capability and enhanced longevity to ensure maintenance of the population over generations. Thus, in bats, the dependence of colony survival as a whole may depend upon enhanced individual survival and delayed senescence (51). Genetic analyses of several bat species have shown differences in the growth hormone (GH)/insulin-like growth factor 1 (IGF1) axis which in humans is associated with aging, resistance to diabetes and cancer (52).

The determinants of adult survival in bats have been historically difficult to identify, as this requires tracking individuals over many years, and until recently longitudinal studies of bat mortality were conducted using tagged bats, of which only a fraction were recovered (53). Recently, a 19-year study of a colony of Bechstein's bats demonstrated that unlike terrestrial mammals, survival could not be predicted by common indicators such as season, age, and body size. Instead, the only accurate predictor of mortality was a single cataclysmic weather event that affected multiple countries in north-central Europe. Additionally, even the oldest female bats were reproductively capable, indicating that bat survival is primarily affected by catastrophic natural events rather than factors that normally dictate an individual's fitness (45).

### Echolocation

Molecular phylogenetic studies of bats suggest that there are massive gaps in bat fossil records. As bats are the second most diverse order of mammals, outnumbered only by rodents, the number of species unrepresented in the fossil records is staggering. Over half of microbat and nearly all of megabat fossil histories are missing (31, 54). The enormous incompleteness of the fossil records has made it difficult to identify when specific morphological traits of bats arose. As molecular phylogeny groups two echolocation-reliant microbat species with megabats (also called Old World bats or pteropodids), which do not rely on echolocation, there is some debate as to whether echolocation first arose in the common ancestor of bats and was subsequently lost in megabats, or whether it arose twice, independently (31). Pteropodids have adaptations that enhance visual acuity at night (55), and they do not require echolocation for foraging (56). There are multiple types of echolocation that can be partially delineated by species, but are more clearly categorized by the type of environment. Divergent species that inhabit the same type of environment, such as those that hunt in large, open spaces, often use the same form of echolocation, suggesting that habitat has a greater influence on echolocation than phylogeny (31). Importantly, echolocation can result in the production of droplets or small-particle aerosols of oropharyngeal fluids, mucus, or saliva, thus facilitating transmission of viruses between individuals in close proximity (57, 58). The unique navigation tactic of many bat species may inadvertently facilitate virus transmission among bats in the same habitat.

### Flight

Bats are the only mammal capable of powered flight, which likely evolved ~65 million years ago alongside birds following radical ecological changes that resulted in the extinction of the dinosaurs (54, 59). During flight, bats consume approximately four times as much oxygen, and they have a markedly higher concentration of red blood cells compared to small terrestrial mammals (60). Bat flight is markedly different from that of birds and insects, whose wing surfaces are typically composed of inflexible material, such as feathers or chitin. Bat wings are constructed from live skin stretched across elongated arm and finger bones, making them extraordinarily malleable and sensitive to environmental cues (59). The plasticity of bats' wings allows them to navigate and inhabit diverse ecospheres, contributing to their extensive speciation. Moreover, the capability of powered flight can allow the efficient spread of viruses and thus the introduction of pathogens to which colonies may otherwise have remained naïve.

As flight is extremely metabolically demanding, in addition to evolving the physical mechanisms required for flight, bats have also evolved necessary underlying molecular mechanisms. The mitochondrial respiratory chain accounts for nearly all ATP required for mobility in eukaryotes, and genetic analysis of both micro- and megabat species revealed an enrichment of genes specific to the oxidative phosphorylation (OXPHOS) pathway. Specifically, 4.9% of nuclear-encoded and 23% of mitochondrial OXPHOS genes have evidence of positive selection in bats, which is markedly higher than the expected 2% of orthologous genes in previous genome-wide studies that show evidence of positive selection (61). Genomic analysis of *Pteropus alecto* and *M. davidii* suggests positive selection for the DNA damage checkpoint pathway and changes in overlapping aspects of this pathway with the innate immune system, indicating that evolutionary adaptations important for flight may have secondarily affected bat immunity (62).

### Social interaction and communities

As a group, bats exhibit the greatest diversity of social systems in mammals. Tropical species are primarily responsible for this diversity, as temperate species are more restricted in their social behavior. Generally, however, bats are extremely social creatures that tend to form dense roosting colonies (63), and almost all temperate-zone species live in closed societies with very little infiltration of foreign bats into established roosts (63, 64). In particular, female bats form maternity colonies in which males do not take part. As bats are capable of long-distance flight, dispersal barriers cannot explain the philopatry of females. Instead, benefits such as knowledge of foraging areas and social thermoregulation likely selected for these colony types. Additionally, there is evidence that forming closed societies limits the potential invasion of new pathogens, thereby protecting colony members that would otherwise be vulnerable to infection. For example, *Pseudogymnaoscus destructans* has decimated North American bat populations that do not live in the type of closed societies observed elsewhere (64). DNA analysis of a closed society of Bechstein's bats revealed extraordinarily high conservation of mitochondrial DNA and relatively low conservation of nuclear DNA, suggesting stable maternal populations within colonies and gene flow between colonies via promiscuous mating with males. It is possible that the mating patterns of temperate-zone species may allow transmission of pathogens between colonies via traveling males while the more insular females may allow viruses to persist throughout generations within a colony.

## Anti-viral immune responses of bats

An important commonality among pathogenic RNA viruses in humans presenting with disease is that the host response is an important contributor to the disease process, with dysregulated and excessive innate immune responses being particularly important drivers of tissue damage during infection (8). Given the general absence of clinical signs of disease in bats infected with the same viruses that are so lethal in humans or other non-natural hosts infected experimentally, a critical question has been to understand whether bats might establish effective disease tolerance, thus maintaining fitness despite pathogen replication, or whether bats are more resistant to infection through more successful control of pathogen replication and what the contribution of the immune response is (65, 66). The lack of many fundamental immunological tools enabling the probing of bat immune responses has meant that truly mechanistic studies of bat immunity have been very limited, although recently there has been some progress in establishing approaches such as flow cytometry to identify distinct bat immune cell populations (67, 68). So far, studies of bat immunity have primarily taken one of three approaches, whereby each comes with important strengths and weaknesses that have to be kept in mind: (i) comparative genome studies, (ii) *in vitro* cell culture assays, and (iii) experimental infections.

Comparative genome studies have confirmed that the critical components of the innate and adaptive immune system are conserved in bats at the gene level and that bats have the machinery for innate responses to pathogen-associated molecular patterns (PAMPs), the production of anti-viral effector molecules such as type I interferons (IFN), T cell responses (variable T cell receptors, MHCI and MHCII), and B cell responses [reviewed in (35)]. Interestingly, based on the 10 bat genomes sequenced so far, the only family of genes lost entirely in all of them are PYHIN genes (69). Members of the PYHIN family are DNA sensors capable of recognizing foreign DNA, including DNA viruses and damaged self DNA which can be generated by RNA viral infection. Recognition of DNA results in production of IFN through interaction with stimulator of interferon genes (STING). The PYHIN family also encode the only identified class of DNA sensors capable of activating the inflammasome. It has been hypothesized that the absence of the PYHIN family may allow bats to limit activation of the innate immune response to damaged self-DNA generated by RNA viral infection, thus avoiding excessive inflammation (69, 70). Genome comparisons highlighting contractions or expansions of specific gene families, specific genes under positive selection, or non-conserved sequence differences in critical protein domains can thus provide the basis for hypotheses worth testing further. However, it is important to note that much can be missed in absence of data on gene regulation, especially during infection when gene expression kinetics can make a critical difference to the infection outcome. Moreover, the absence of a gene or gene family does not rule out that other proteins have evolved to compensate for their loss of function. Thus, while whole genome analyses can provide a context for specific questions or be hypothesis-generating, on their own they cannot distinguish tolerance from resistance mechanisms. The repeated identification of signatures of positive selection in innate immune genes in particular, does however lend credence to the idea that bats have specific adaptations as a result of a long co-evolutionary history with viruses.

Cell culture assays with bat cell lines, or, in some instances, primary bat cells, have been used to assess whether bats are permissive for viral replication and to determine whether particular immune receptor signaling pathways are intact. As discussed below, such studies have probed the type I IFN pathway in particular, revealing some possible species-specific differences among bats (71–83). However, it is important to note that in some instances immortalized cells can behave differently from primary cells and that such cultures may miss additional differences imposed by changes in cell localization, cell recruitment or cell-cell interactions in a whole animal. Careful experiments measuring the quality, magnitude, and kinetics of immune responses in bats during infection and upon administration with defined stimuli for which we have comparative information from humans remain to be done to provide additional evidence that specific innate immune pathways are wired differently.

Experimental infections come with the enormous challenge of having to house and/or breed colonies of bats and to have biosafety-level 4 facilities in place to perform infections with viruses lethal to humans. Moreover, some trial and error is involved in determining which route and dose leads to viral replication, establishing a source of the virus (human-adapted strains tend to replicate less well in bats than strains obtained from naturally infected bats), and amplifying this viral stock without extensive tissue culture passaging. Studies to date have examined the kinetics of viral replication by quantifying the extent of viremia and dissemination to other tissues, and assessing changes in white blood cell counts, body mass, and temperature. Given the generally low levels of viral shedding and short infectious periods observed so far it remains poorly understood how transmission occurs in the wild to sufficient levels that cross-species jumps occur. Some infection experiments have also provided evidence that a particular bat species is unlikely to be a reservoir despite epidemiological evidence, for example for *R. aegyptiacus* and ebolavirus. Certainly, once good experimental infection models are established, such studies have the potential to be hugely informative with regard to anti-viral immune responses elicited using, for instance, comparative transcriptome analyses. One drawback may be that experimental infections do not mimic the impact of chronic stress arising from the disruption of wildlife populations, which bats are particularly sensitive to Jones et al. (84). Comparison of either cave-roosting or foliage-roosting species in areas of Malaysian Borneo designated as actively logged forest, recovering forest, or fragmented forest revealed varying impacts of habitat disturbance on stress and circulating white blood cells (85).

Overall, the limited studies of bat immunity that have been done have focused largely on 2 species: *P*. *alecto* and *R. aegyptiacus*. We summarize this work below, but comparisons of observations made across species suggest that although a number of species appear to be capable of avoiding the pathological effects of RNA virus infection, each bat species may have achieved this through distinct pathways, possibly involving changes to both increase pathogen replication control and to mitigate any immunopathology through decreased inflammatory responses and hence increased disease tolerance.

### Pteropid bats

The most well studied bat species with regard to antiviral immune responses is the Australian black flying fox (*P. alecto*). This interest has stemmed from the fact that pteropid bats have been identified as the natural reservoirs for the deadly Hendra and Nipah viruses (86), which continue to cause outbreaks [such as most recently in India in May 2018 (87)]. To date, several studies have examined the kinetics of viral infection in Pteropus bats and the nature of transmission and replication in other susceptible species (88–91). In Australia, all four species of pteropid bats (*P. alecto, P. poliocephalus, P. scapulatus*, and *P. conspicillatus*) have antibodies to Hendra virus but only *P. alecto* and *P. conspicillatus* are considered to be the primary reservoir hosts (14, 92, 93). In South East Asia, both pteropus spp. occurring in Malaysia have been found to be seropositive for Nipah virus neutralizing antibodies, and the virus has been isolated from *P. hypomelanus* and *P. vampyrus* (15, 94).

Experimental infections of pteroid bats with Hendra or Nipah virus result in sub-clinical infection with short periods of virus replication and shedding, and low antibody titres (88–91). Upon subcutaneous infection of *P. poliocephalus* with Hendra virus, viral antigen was detected by immunohistochemistry at 10 dpi in blood vessels of spleen, kidney and placenta (89). Similarly, oronasal Hendra virus infection of *P. alecto* led to the presence of viral genome in lung, spleen, liver and kidney 3 weeks later, but virus isolation was unsuccessful at this timepoint (89, 91). The Malaysian flying fox, *P. vampyrus* and the Australian species, *P. poliocephalus* demonstrate similarly short periods of viremia upon infection with Nipah virus. In subcutaneously infected *P. poliocephalus*, virus was isolated from the kidney and uterus of bats euthanized at 7dpi, but no virus was isolated at any of the other timepoints examined (3, 5, 10, 12, or 14 dpi) and there was no evidence of antigen in any tissue by immunohistochemistry, including tissues collected at 7 dpi. In this study, low neutralizing antibodies were detected in all bats with the exception of one individual that developed a significant neutralizing antibody titre — possibly reflecting the fact that *P. poliocephalus* is not the natural host for Nipah virus (90). In *P. vampyrus* challenged by oronasal Nipah inoculation, viral genome was detected in a throat swab at 4 dpi and a rectal swab of the same individual at 8 dpi but virus was undetectable in tissues collected at post-mortem from all individuals (49, 50, or 51dpi), consistent with a short period of viremia. Similar to previous studies, antibody titres were low in all *P. vampyrus* bats (91). Overall, these results are consistent with bats controlling replication rapidly, at least following experimental infections which involve higher doses of virus compared to what bats would likely be naturally exposed to in the wild. The absence of a robust antibody response also appears to be typical of all experimental Hendra and Nipah virus infections performed to date. Since antibody responses are the only immune parameter that has been measured during experimental infections of bats so far, it is difficult to speculate on the mechanisms responsible for control of viral infections *in vivo*.

*Pteropus alecto* was among the first bat species to have its genome described in detail. Genomic studies provided initial clues for possible differences in the innate immune system of bats, with evidence for selection of key innate immune genes and the expansion or contraction of specific immune gene families (62, 68, 95). The MHCI region is contracted (96), as is the type I IFN locus, which in *P. alecto* contains fewer IFN genes than any other mammalian species sequenced, with only three functional IFN-α loci (68). In contrast, pteropid bats have the largest and most diverse family of APOBEC (apolipoprotein B mRNA editing enzyme, catalytic polypeptide-like) proteins identified in any mammal (95). APOBECs interfere with the replication of retroviruses by deaminating cytosine residues in nascent retroviral DNA. This is notable, as bats are an important source of mammalian retroviruses, many of which have been transmitted to other mammals (97, 98). APOBEC diversification may therefore have occurred to counteract the effect of retroviruses and possibly other viruses, as APOBECs have been shown to restrict the replication of other virus families including hepadnaviruses, and parvoviruses (99, 100). Members of the APOBECA3 protein family exhibit direct antiviral activity through DNA cytosine deamination which results in hypermutation of the nascent retroviral DNA which is then degraded or rendered non-functional (101). The mechanism of antiviral activity against non-retroviruses remains largely unknown. For parvovirus adeno-associated virus, APOBEC meditated inhibition has been speculated to involve direct interaction with the viral DNA or the replication machinery (102). Whether the expanded family of ABOBECs in bats have evolved other mechanisms to control DNA and RNA viruses remains to be determined. As APOBECs can be induced by even low levels of type I IFN (103), one hypothesis to be tested is that bats, through their multiple APOBECs, are able to restrict viral replication without causing inflammation. *Pteropus alecto* is the only bat species to date in which APOBEC genes have been mapped, and whether the expansion of this gene family extends to other bat species remains to be determined.

In addition to the identification of putative immune pathways distinct in *P. alecto* through genome studies, differences have been identified in the activation of innate immune effectors in *P. alecto* from studies performed *in vitro*, primarily using cell lines derived from tissues including the kidney and lung. IFNs are the first line of defense following viral infection and unsurprisingly, because of this, they have been the most extensively studied group of genes in bats. Both type I (IFNA and IFNB) and III (IFNL) IFNs are detectable in bat cells. Curiously, a unique characteristic of pteropid bats is the constitutive expression of mRNA for IFNA and the signaling molecule, IFN regulatory factor 7 (IRF7) in unstimulated tissues and cells [75, 68a]. Constitutively expressed IFNA and IRF7 may allow bats to respond more rapidly to infection, thus avoiding the lag time between pathogen detection and response. Furthermore, viral infection or stimulation with synthetic ligands result in little IFNA induction in pteropid bat cells (68). The constitutive expression of IFNA has been described in two species of pteropid bats (*P. alecto* and *Cynopterus brachyotis*) and is a first for any species. IFNB and IFNL are activated following stimulation of cells from *P. alecto* and *P. vampyrus* with synthetic ligands such as polyIC (71–74). Moreover, bat IFNs demonstrate antiviral activity (68, 71–74, 104). However, viral infection of *P. alecto* splenocytes results in induction of IFNL but not IFNB, hinting at differences in the function of type I and III IFNs (74). In humans and mice, IFNL has recently been demonstrated to have a role not only in controlling virus replication, but also in dampening damage-inducing neutrophil functions and in modulating tissue-damaging, transcription-independent responses such as production of ROS (77, 80). A hypothesis yet to be tested is whether upregulation of IFNL rather than IFNB has a similar function in bats.

The endoplasmic reticulum (ER) membrane protein, STING, is involved in induction of type I IFN by cytosolic DNA (105). Stimulation of bat splenocytes with GMP-AMP, which is produced following sensing of cytosolic DNA by cGAS, results in little induction of IFN compared to responses observed in mouse splenocytes (83). Bat STING contains an amino acid substitution of the highly conserved and functionally important serine residue S358 which may be responsible for dampening STING-dependent IFN activation in bat cells in response to DNA. However, comparable levels of IFN induction in mouse and bat cells in response to the RNA viral mimic polyIC indicate that STING-associated inhibition of the IFN response does not extend to RNA viruses (83), thus the relevance to RNA viruses in bats remains unknown.

Downstream of the induction of IFNs, novel subsets of IFN stimulated genes (ISGs) have been detected in unstimulated and stimulated pteropid bat cells indicative of a response that is less damaging to the host. Furthermore, the ISG response is elevated for a shorter period of time in *P. alecto* compared to human cell lines which again may be a strategy to avoid tissue damage (78, 81). The less inflammatory profile of ISGs may be the key to the ability of bats to tolerate higher IFN expression without adverse consequences. The balance between resistance and tolerance may therefore be achieved through careful selection of the pathways that are activated and shorter periods of activation or limited activation to prevent inflammation. In this regard, studies of the regulation of IFN signaling in bats is likely to provide important additional insights.

### Rousettus bats

A second bat species whose host responses to viral infections has been studied more recently is the Egyptian fruit bat (*R. aegyptiacus*). Marburg virus (MARV) has been repeatedly isolated from this species with demonstrated seasonal pulses of active MARV replication in juvenile bats living in caves in Uganda (11, 106). Moreover, *R. aegyptiacus* were a suspected reservoir for ebolavirus (EBOV) based on epidemiological evidence and detected seroreactivity to EBOV, but no infectious virus has been isolated thus far from wild rousettus bats (107). Indeed, while cell lines from *R. aegyptiacus* are equally susceptible to MARV and EBOV (79, 108), experimental infections of *R. aegyptiacus* seem to confirm that it is a reservoir for MARV, but is unlikely to be the source of EBOV spillover to humans. Subcutaneous EBOV infection results in very low viral replication, no viremia, little dissemination to other tissues, and no viral shedding, although some animals seroconvert, suggesting that *R. aegyptiacus* are unlikely to perpetuate EBOV in the wild (109, 110). In contrast, experimental MARV infection of *R. aegyptiacus* resulted in acute viremia that peaked on days 5–6 post-infection (although generally at lower levels than in humans), oral shedding that peaked on days 7–8 post-infection, and dissemination to other tissues including spleen, liver, kidney and salivary glands (109, 111–113). Interestingly, viral replication was not associated with increases in white blood cell counts, any clinical signs of infection such as changes in body temperature or body weight, and infected tissues showed little evidence of inflammatory infiltrates (109). In all experiments, viremia was cleared by day 13 and oral shedding ceased by day 19. Intriguingly, a cohousing experiment resulted in MARV transmissions to uninfected bats 4–7 months after experimental infection, raising the question of whether persistent infection with intermittent shedding is possible or whether very long latent periods without detectable viral replication could follow exposure (114). Upon secondary challenge of previously MARV-infected bats, none showed any detectable viral replication or shedding, providing evidence that protective immunity is established (115).

Unlike for pteropus bats, no constitutive expression of type I IFNs has been detected in *R. aegyptiacus* (79), but type I IFNs are induced in *R. aegyptiacus* cell lines upon stimulation with Sendai virus as seen in other mammals (82). Furthermore, in *R. aegyptiacus* the type I IFN genes are expanded, again in contrast to *P. alecto* (82), but like for *P. alecto* a number of genes in the type I IFN pathway or involved in innate immune recognition of PAMPs show signs of having been under positive selection (82). Whether positive selection of genes in either bat species is associated with tolerance remains to be determined, especially given that innate immune genes in humans have also been under positive selection (116). A transcriptome study which generated 20 RNA sequencing libraries from 11 tissues taken from 1 female and 1 male *R. aegyptiacus* found a reduced coverage of NK cell related genes compared to other mammals, but confirmed that in these bats the predominant T cells had an αβ T cell receptor, and showed that IgE, IgG, IgM, and IgA, as well as a number of pro- and anti-inflammatory cytokines, were all detectable (117). The recently sequenced *R. aegyptiacus* genome revealed substantial differences in the repertoire of NK cell receptors, with this bat species entirely lacking functional killer cell immunoglobulin receptors (KIRS) and with all killer lectin-like receptors (KLRs) encoding either activating and inhibitory interaction motifs, or inhibitory interaction motifs only (82). NK cells are important immune cell players in an antiviral response but without assessment of the consequences of these genomic differences it is difficult to draw any specific conclusions with regard to viral control or the magnitude of inflammation elicited upon infection with viruses like MARV. Nonetheless, these genomic data provide some interesting hypotheses to be tested in the future.

### Other bat species

Some additional studies probing the induction of cytokines upon stimulation of bat cells with defined innate immune stimuli provides some evidence that innate immune recognition of viruses may be altered, leading to a reduction in pro-inflammatory responses. Stimulation of kidney and myeloid cells from the big brown bat (*Eptesicus fuscus*) with polyinosinic-polycytidylic acid (polyI:C) resulted in only limited activation of the inflammatory cytokine, tumor necrosis factor alpha (TNFα) compared to human cells which display a robust TNFα response. Induction of TNFα is controlled by transcription factors, including the NF-kappa B (NF-κB) family which consists of five members, [RelA (p65), RelB, c-Rel, NFκB-1 (p50), and NFκB-2 (p52)] which form homo- or hetero-dimers that are bound by molecules of the inhibitor of NFκB (IκB) family and retained in the cytoplasm of the cell in an inactivated state (118). In *E. fuscus*, a potential repressor (c-Rel) binding motif was identified in the TNFα promoter region which may explain the difference in induction of TNFα in *E. fuscus* cells. Consistent with this hypothesis, partial knockdown of c-Rel transcripts significantly increased basal levels of TNFα transcripts in *E. fuscus* cells (104). The transcription factor, c-Rel has also undergone positive selection in the bat ancestor which may indicate that this mechanism is common to other species of bats (62). Of note, low levels of TNFα induction have also been associated with tolerance in European bank voles which are a natural reservoir for Puumala hantavirus (PUUV) (119).

Stimulation of macrophages from the greater mouse eared bat (*Myotis myotis*) suggested that this species may have also evolved mechanisms to avoid excessive inflammation caused by cytokines. While high levels of TNFα, IL1β, and IFNβ were produced in response to *in vitro* challenge with lipopolysaccharides (LPS) and PolyI:C, there was also a sustained, high-level transcription of the anti-inflammatory cytokine IL-10, which was not observed in mouse macrophages (120). Furthermore, unlike in the mouse, *M. myotis* macrophages did not produce the proinflammatory and cytotoxic mediator, nitric oxide, in response to LPS. The same study also showed evidence of bat specific adaptations in genes involved in antiviral and pro-inflammatory signaling pathways through comparison with other mammalian taxa, including RIG-I, IL1b, IL-18, NLRP3, STING, and CASP1, further supporting the evolution of adaptations associated with reducing inflammatory responses in bats (120).

## Bat immune responses to non-viral pathogens

Even less is known about immune responses of bats to non-viral pathogens than to viral pathogens, but it is clear that while anti-inflammatory responses may be characteristic of anti-viral responses in bats, they are susceptible to disease upon infection with particular pathogens—in some instances due to dysregulated and damaging immune responses. One particular example of this is the emerging infectious disease, white nose syndrome (WNS), that has decimated North American bat populations beginning in 2006, in what will likely rank as one of the most devastating wildlife diseases in history (121–123). For reasons that remain poorly understood, the psychrophilic fungus *Pseudogymnoascus destructans* (formerly *Geomyces destructans*) causes no mass mortality in European bats despite being abundantly detected (124, 125). Indeed, evidence suggests that a single *P. destructans* genotype was introduced to North American bat species from Europe (125). In North America, *P. destructans* infection is not specific to a particular bat genus, replicating in many different bat species during hibernation and targeting the furless skin of the wings, ears, and muzzle (126). Distinct hypotheses have been proposed for why *P. destructans* is so deadly in North American bats, ascribing the impaired tolerance to infection compared to European bat counterparts to either physiological or immunological factors. On the one hand, more frequent arousal, electrolyte depletion, and dehydration are thought to contribute to mortality following infection (127, 128). The destruction of wing tissue in WNS results in a marked electrolyte imbalance, as the wings play a critical role in maintaining water levels, especially during hibernation, during which bats are particularly vulnerable to dehydration (129, 130). Dehydration catalyzes arousal in hibernating bats, which is extraordinarily metabolically costly and rapidly depletes the fat reserves necessary to survive until spring (127). An alternative hypothesis posits that the restoration of the immune system following emergence from hibernation induces the fatal pathology of WNS. During hibernation, destruction of cutaneous tissue is limited and infiltrating immune cells are entirely absent, yet in the weeks following arousal, infected bats exhibit overt wing damage and corresponding neutrophilic and lymphocytic infiltration (131). Hibernation does not preclude a localized immune response to *P. destructans* at the site of infection and transcriptomic analysis of infected tissue showed upregulation of some acute inflammatory genes in infected tissue (132, 133). However, the observed immune responses likely occur during arousal periods, which are more common in infected bats. Ultimately, immunosuppression during torpor allows *P. destructans* to colonize infected bats relatively unchecked (124), and upon emergence from hibernation, the exuberant immune response may result in deadly immunopathology during WNS (131).

In addition to general studies of immune cell recruitment and transcriptional responses during WNS, body mass and white blood cell counts were examined following LPS administration in four bat species (134–137). Subcutaneous LPS challenge in of Pallas's mastiff bats (*Molossus molossus*) led to a loss of body mass of ~7% within the first day, but did not result in changes in circulating white blood cell counts or body temperature (135). Seba's short-tailed fruit bat (*Carollia perspicillata*) also showed a decrease in body mass following LPS challenge, but this was associated with increases in white blood cell counts as well as increases in derivatives of reactive oxidative metabolites (dROM) (134). Subdermal LPS challenge of fish-eating Myotis (*Myotis vivesi*) led to body mass decreases, increased resting metabolic rate and skin temperature (136), while intraperitoneal LPS challenge of wrinkle-lipped bats (*Chaerephon plicatus*) caused an increase in circulating leukocytes, but did not result in a reduction in body mass compared to controls (137). The differential responses to LPS challenge suggest that the immune response to bacterial infection varies across species. Of note, post-mortem examinations of ~500 dead bats comprising 19 species from Germany revealed inflammatory lesions, many of which had evidence of underlying bacterial or parasitic infections, particularly in the lung (138).

## Conclusions

Bats have an array of unique life history characteristics that not only allow them to be particularly good reservoirs for viruses that are highly pathogenic in other species, but also appear to have shaped their immune systems. Although research on bat antiviral immunity has focused on only a few species to date, at the genomic level, selection on genes is concentrated on the innate immune system across both suborders of bats. However, while these studies have provided a rich source of hypotheses, the majority remain to be tested at the functional level and many questions remain that cannot be answered from comparative genome studies. Experimental studies to date have demonstrated some functional differences between bat species, with the common emerging theme that the overall antiviral response appears to converge on a lower inflammatory profile, with tight regulation of the cytokine and inflammatory response key to clearing viral infection without the pathological outcomes typically associated with infection. However, whether this is due to specific tolerance mechanisms that are at play or increased resistance to RNA virus replication still remains unclear. Fewer studies have examined the adaptive immune system than those probing innate immune pathways, but experimental infections with bat borne viruses have demonstrated that bats generate low or absent antibody responses which often wane rapidly. This is reminiscent of the response of another reservoir host, the sooty mangabey which is the natural reservoir for simian immunodeficiency virus (SIV) and for yellow fever virus. Sooty mangabeys given an attenuated yellow fever virus vaccine strain generate much lower, transient antibody responses as compared to humans or rhesus macaques. Changes to innate immune responses are also evident in sooty mangabeys (139). Thus, intriguingly, different reservoir hosts may have arrived at similar solutions to avoid the pathological consequences that follow viral infection in non-natural hosts.

Despite the ability of bats to avoid disease associated with viral infection, this trait does not extend to all pathogens, as evidenced by the severe consequences associated with infection of North American bats with the fungus that causes WNS. Thus, the pathways associated with the control of other pathogens have not been under the same selection pressures as those responsible for controlling infections with RNA viruses—or there are immunological trade offs involved which lead to greater susceptibilities to some pathogens than others. Overall, it is clear that studying host-pathogen interactions in reservoir hosts has considerable potential to provide novel insights into host tolerance mechanisms that eventually could assist in the treatment of diseases in humans and other susceptible hosts and may also offer solutions for the treatment of diseases that are a conservation threat to bats themselves.

## Author contributions

All authors listed have made a substantial, direct, and intellectual contribution to the work and approved it for publication.

### Conflict of interest statement

The authors declare that the research was conducted in the absence of any commercial or financial relationships that could be construed as a potential conflict of interest. The handling Editor declared a shared affiliation, though no other collaboration, with the authors JM and CS.
